# Measles outbreak investigation in Berhet District, North Shewa, Ethiopia

**DOI:** 10.3389/fpubh.2024.1330205

**Published:** 2024-05-02

**Authors:** Yohannes Shimelis, Anemaw Asrat, Tesfahun Tadege, Sefineh Fenta Feleke

**Affiliations:** ^1^Department of Epidemiology and Biostatistics, School of Public Health, College of Medicine and Health Sciences, Bahir Dar University, Bahir Dar, Ethiopia; ^2^Amhara Public Health Institute, Bahir Dar, Ethiopia; ^3^Department of Public Health, College of Health Sciences, Woldia University, Woldia, Ethiopia

**Keywords:** measles, outbreak, Berhet district, Northshewa, Ethiopia

## Abstract

**Introduction:**

Measles, though usually self-limiting, can have severe consequences influenced by factors such as vaccination and nutrition, notably vitamin A deficiency and malnutrition. Despite progress, contextual changes and implementation issues have hampered efforts, resulting in increased outbreaks and cases of measles. This study seeks to pinpoint outbreak features, risk factors, and strategies for preventing and controlling measles.

**Methods:**

A descriptive cross-sectional study and a 1:2 unmatched case-control study design were employed. All 101 suspected measles cases listed on the line-list were included in the descriptive research, with 60 measles patients and 120 controls included in the case-control investigation. Line-list data were cleaned and analyzed using a pivot table in Microsoft Excel 2016. Subsequently, the data were cleaned, entered into Epi Info 7.2, and exported to SPSS 26 for analysis.

**Results:**

Twenty cases occurred per 10,000 individuals. Men accounted for 67.3% of cases, with ages ranging from 5 months to 45 years and mean and standard deviations of 9.6 and 7.6, respectively. Age group of 5–14 years comprised 57.4% of cases, followed by 1–4 years with 24.8%. Being unvaccinated against measles showed an adjusted odds ratio (AOR) of 12.06 (95% CI: 3.12–46.52). Travel history to regions with active cases had an AOR of 5.73 (95% CI: 1.78–18.38). Contact with a measles patient showed an AOR of 10.3 (95% CI: 3.48–30.5). Understanding the measles transmission mechanism had an AOR of 0.164 (95% CI: 0.049–0.55), and awareness of the disease's preventability had an AOR of 0.233 (95% CI: 0.67–0.811). All factors were independently associated with the illness.

**Conclusion:**

This outbreak affected a broader age range with a high attack rate, mainly in the age group of 5–14-years. Over 35% of cases lacked measles vaccination, indicating low administrative vaccine coverage. Factors contributing to the outbreak include lack of measles vaccination, travel to areas with active disease, contact with cases, and insufficient knowledge of measles transmission and prevention strategies among mothers and caregivers.

## Introduction

The measles virus causes the acute, highly contagious disease known as measles. The measles virus belongs to the Paramyxoviridae family's genus Morbillivirus (1). There is little indication that the viral antigens have altered considerably over time, suggesting that the virus is antigenically stable (2). Nevertheless, viral genome sequencing has revealed that different lineages (genotypes) of wild-type measles viruses exist (3). The discovery of a particular virus genotype, when taken into account alongside epidemiological data, can indicate the source of an outbreak. The measles virus is susceptible to drying, heat, and UV light. The virus can only survive for around 2 h in the air or on surfaces and objects (4). The upper respiratory tract, or the conjunctiva, is the primary site of infection for this extremely infectious virus, which is mostly spread by respiratory droplets or airborne spray. The measles virus only naturally occurs in humans. While monkeys are susceptible to infection, there does not seem to be a mechanism for the virus to spread among them in the wild. Children who visit endemic places or come into contact with travelers who have been there are among the risk factors for measles virus infection, regardless of their vaccination status (5). These include children with vitamin A deficiency and immunodeficiency brought on by HIV or AIDS, leukemia, alkylating drugs, or corticosteroid medication, as well as newborns who lose passive antibodies before the age of normal vaccination. Malnourished people and young children are especially vulnerable to complications and mortality from measles infection (3).

Leukopenia is linked to the incubation phase, which is thought to span 10–14 days. Fever that is accompanied by cough, coryza, and/or conjunctivitis indicates the prodromal phase. The rash typically appears 3–5 days after the fever appears. Before the rash appears, during the prodromal phase, the virus starts to shed. Nectin-4 causes the respiratory epithelium to become basolaterally infected after viremia caused by infected lymphocytes, and viral transmission persists through respiratory secretions. From around 4 days before the development of the rash to 4 days following it, people are thought to be contagious. The entire simple illness course takes 17–21 days, starting at the feverish onset (3, 6).

Although measles recovery results in lifetime immunity, the patient paradoxically undergoes temporary immunosuppression during and after acute infection, which is supported by the suppression of delayed-type hypersensitivity responses (4, 7). The primary cause of measles-related morbidity and mortality is pneumonia or gastrointestinal infections, which are typically brought on by secondary bacterial infections brought on by immunosuppression (8).

Although the measles is self-limiting, several serious consequences have been reported. Morbidity and mortality of measles are complex, influenced by vaccination as well as nutritional status; severe results are associated with deficiencies in vitamin A and malnutrition. There is a rare chance that measles will cause problems with the central nervous system (CNS). Acute disseminated encephalomyelitis and primary measles encephalitis are two of the severe diseases that have been reported (7).

The estimated global measles case burden exceeded 9.7 million cases in 2015, with 254,928 reported cases across all six regions of the WHO, for an estimated total of 134,200 measles deaths (7). One known limitation of measles case surveillance is that it is subject to underreporting. Studies have demonstrated underreporting in outbreak settings (7).

The WHO regions with the highest incidence were those in Africa, the Eastern Mediterranean, and Europe in 2014–2015, as a result of significant measles outbreaks. The regions with the highest number of cases reported were Africa (98,621; incidence of 100/million), the Americas (423; 0.6/million), the Eastern Mediterranean region (21,335; 33/million), the European region (25,974; 31/million), South-East Asia (29,109; 17/million), and the Western Pacific region (65,176; 35/million) (4, 7). The region of the Americas verified the elimination of measles in 2016, demonstrating the feasibility of elimination in low- and middle-income countries (1).

Every Member State in all six WHO Regions has set goals to eradicate measles by 2020 or earlier, and the WHO Global Vaccine Action Plan for 2012–2020 has set the target of eliminating measles and rubella in at least five WHO Regions by 2020 (9). Despite ongoing viral incursions from other parts of the world, the Region of the Americas was able to achieve and maintain the eradication of endemic measles transmission in 2002, lasting for over 10 years (10, 11). This outstanding accomplishment has inspired efforts to attain elimination in other regions and resulted in many lessons being learned. The Americas are currently setting the standard for measles prevention, having been the first region to eradicate polio. The event has also brought attention to the continuous difficulties in maintaining elimination; measles is a global issue that affects everyone (11).

Over 11 years (2004–2014), 7,296 samples were collected in Amhara Regional State, with 2,412 (36.7%) testing positive for measles IgM. Those aged 10 years and above were most affected, and in 2014, all 11 zones reported cases, with a peak during the hot, dry season (9). This study aims to investigate the measles outbreak in the Berhet district and North Shewa zone of Ethiopia's Amhara region in 2022.

## Methods and materials

### Study area and period

**Figure 1:**
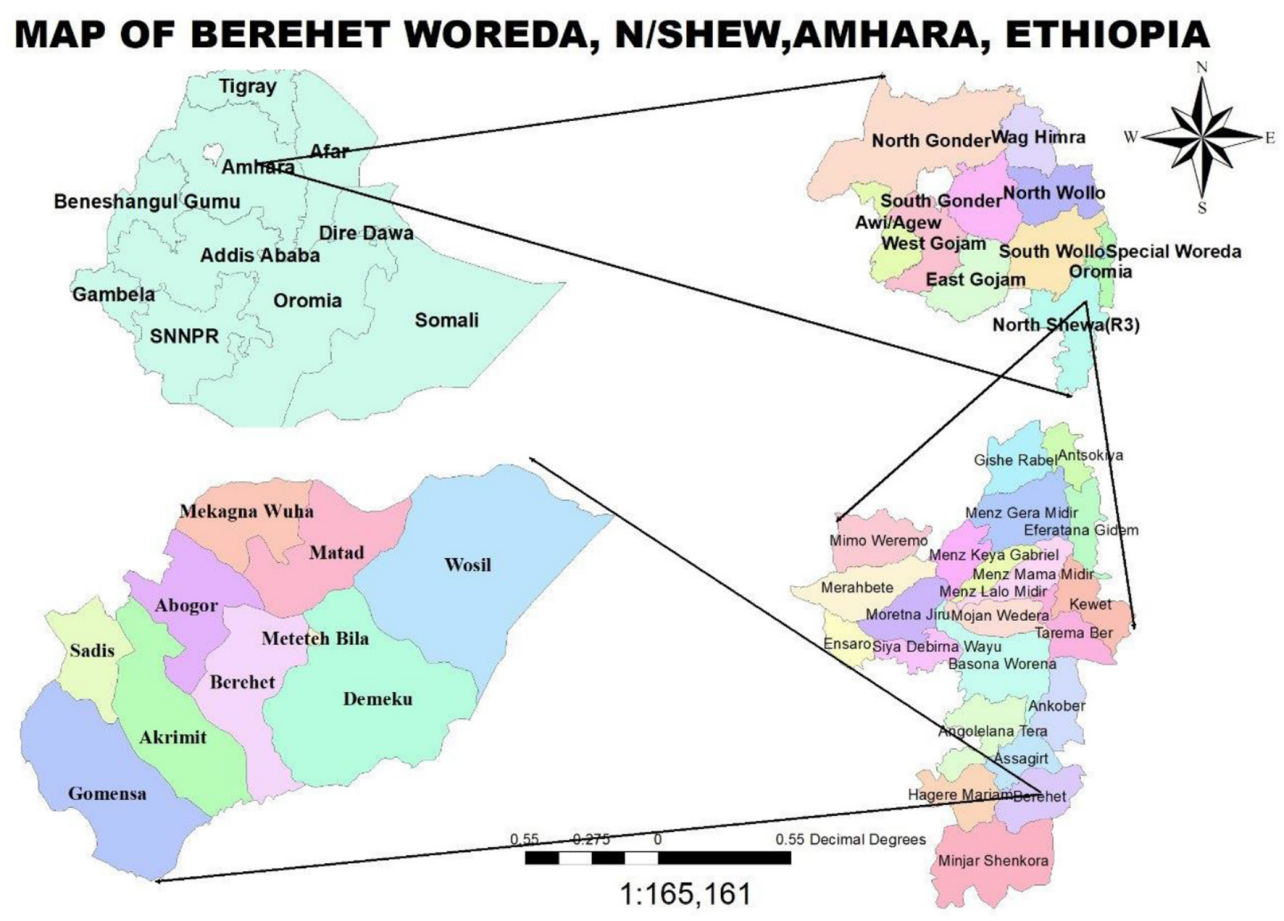
Map of the study area.

The research was conducted in Berehet, one of the 24 districts in North Shewa, Amhara, Ethiopia. It is located 230 km from Addis Ababa, Ethiopia's capital, 830 km from Bahir Dar, the Amhara Region's seat, and 145 km from Debre Berhan Town, which serves as the capital of the North Shewa Zone. The Woreda has a population of 45,349, comprising 20,472 men, 18,723 women, 4,626 individuals aged <5 years, and 1,528 children aged <1 year. Berehet Woreda shares borders with Asagirt Woreda to the north, the Afar region to the east, Haile Mariam Kesem Woreda to the west, and Minjar Woreda and Oromia region Fentalie Woreda to the south. The Woreda consists of nine rural and nine urban kebeles, four health centers, nine health posts, and three medium-sized private clinics. Health posts provide outreach services, while health centers in the district offer static immunization services. The data collection period for the descriptive study was from 13 February 2022 to 20 May 2022. For the case-control study, data for the cases were collected from 4 April 2022 to 10 April 2022 and for the controls from 6 May to 16 May 2022, after the two incubation periods for the cases under investigation (Figure 1).

### Study design

We utilized an unmatched case-control study design at a ratio of 1:2 alongside a descriptive cross-sectional study.

### Sampling procedures and techniques

The descriptive research included all 101 suspected measles cases that were listed on the line-list. For the case-control study, 60 cases were listed throughout the investigation. We used all 60 cases and 120 controls.

### Operational definitions

During the outbreak investigation, we used suspected, confirmed case, epidemiologically linked cases, and measles-related death definitions (12).

*Suspected case:* A person with fever and maculopapular (non-vesicular) generalized rash and cough or coryza (runny nose) or conjunctivitis (red eyes).

*Confirmed case:* A possible case that has been confirmed by testing (positive IgM antibody).

*Epidemiologically linked case:* Any unconfirmed case of suspected measles that is linked (by person, place, and time) to a laboratory-confirmed case; that is, living in the same or neighborhoods where a laboratory-confirmed case is located and where there is a chance of transmission; the rash onsets in the two cases happening within 30 days of each other.

*Measles-related death:* It is a death that happens in a person who has been diagnosed with confirmed measles and that is not related to any other illness, such as trauma or accident, and happens within 30 days of the rash starting.

*Kebele:* The smallest administrative unit in Ethiopia.

### Variables of the study

#### Dependent variable

Measles case (Yes/No).

#### Independent variable

Socio-demographic factors (age, sex, marital status, educational status of the client and mother, and occupational status), vaccination status, contact history, travel history, knowledge of the mode of transmission of measles, and knowledge that measles is vaccine-preventable.

### Inclusion and exclusion of cases and controls

*Cases:* Individuals living in the Berhet district who exhibit clinical indications and symptoms consistent with a proven case, as defined by the national measles guidelines; these cases can be laboratory-verified, suspected, or epidemiologically linked to confirmed cases. Those on paper who did not meet the criteria for a suspected or confirmed case were excluded.

*Controls:* People who live in the same neighborhood as the case but do not meet the criteria for a measles case in Bereket.

Excluded individuals were those who had a known history of measles disease.

### Data collection methods

In the line-listing used for the descriptive study, we identified cases of measles. The national standard case definition was used to identify cases for the case-control study. After locating the cases on the line-list, we interviewed the head of the household, Berhet district health office personnel, health center PHEM focal, and health extension workers. After two incubation periods, data were gathered for controls—those who do not exhibit measles signs and symptoms in the same neighborhood as the case. A structured questionnaire was adopted from the CDC outbreak investigation tool, which comprises three sections (the first section covers socio-demographic information, the second section focuses on clinical features, and the third section explores the associated factors of measles infection). Parents or caregivers were interviewed on behalf of their children if their age was <18 years. Five health personnel participated in data collection, with four health officers as data collectors and one MPH in epidemiology holder as supervisor.

### Data processing and management

For the descriptive study, line-list data were utilized. Microsoft Excel 2016 pivot tables were used to clean, arrange, and analyze the data. The case-control data were cleaned, then imported into Epi Info version 7.2 and exported to SPSS version 26 for further analysis.

### Data analysis

For the descriptive study, line-list data were cleaned, and Microsoft Excel 2016 pivot tables were used for analysis. Descriptive analysis was employed to calculate and compile measles cases based on person, time, and place.

For the case-control study, all categorical variables were cross-tabulated with the outcome variable, and the frequencies and proportions of each variable were reported for the case and control groups. All explanatory variables that were significantly associated with the outcome variable in the bivariate logistic regression at *p* < 0.20 were added to the multivariable logistic regression model to identify independent factors associated with measles infection.

The model's appropriateness was evaluated using the Omnibus test, the Hosmer and Lemeshow goodness-of-fit test, and other methods. The adjusted odds ratios (AORs) and corresponding confidence intervals (CIs) were used to assess the strength of the correlations between the predictor and outcome variables for a *P* < 0.05. To present the results, tables, images, and text were utilized.

### Ethical consideration

An ethical approval support letter was obtained from the Amhara Public Health Institute, which is an autonomous sub-national public health institute. One of the responsibilities of the institute is to provide ethical approval and consent letters for research projects conducted in the region. Additionally, permission was obtained from the Berhet district health office, and we investigated the measles outbreak with documentation from the North Shewa zone health department. After addressing the study objectives and confirming participants' willingness, all involved—participants, parents, or caregivers—provided verbal informed consent, ensuring confidentiality.

## Results

### Descriptive analysis

**Figure 2:**
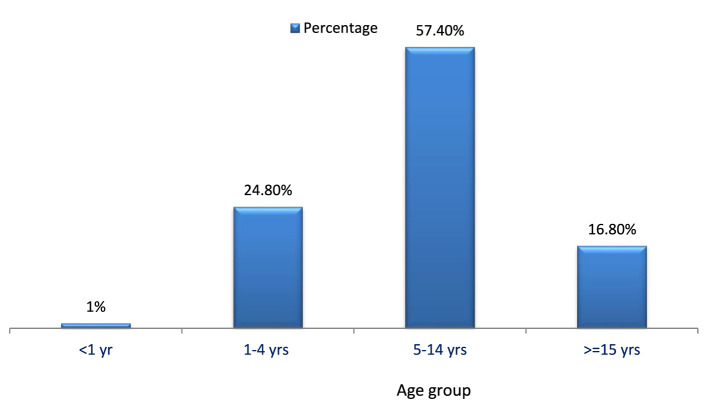
Proportions of measles cases by age group in Berehet district, Amhara region, Ethiopia, 2022.

Between 13 February 2022 and 20 May 2022, a total of 101 measles cases were reported in the Berhet district, North Shewa, comprising three rural kebeles and one urban kebele. No fatalities were recorded. Among the cases, 68 (67.3%) were male, with respective attack rates of 0.3% for men and 0.1% for women. The age of affected individuals ranged from 5 months to 45 years, with a mean of 9.6 years and a standard deviation of 7.6 (refer to Figure 2).

### Description of cases by person

Forty (57.4%) of the cases occurred in the age group of 5–14 years, followed by the age group of 1–4 years (24.8%). No deaths were reported in any age group. The overall attack rate was 20 per 10,000. Most of the cases (67%) were male.

### Description of cases by place

**Figure 3:**
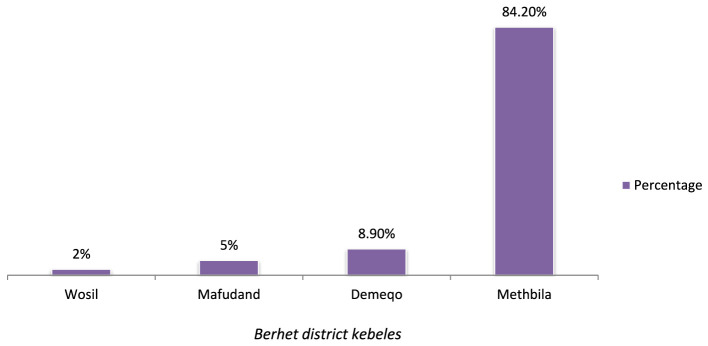
Distribution of measles cases by kebele in Berhet district, Amhara, Ethiopia, 2022.

The cases were reported from four kebeles: Methbila, Demeqo, Mafudand, and Wosil. The majority of cases (85 or 84.2%) were reported from Methbila kebele, which is the urban kebele in the district (refer to Figure 3).

### Description of cases by time

**Figure 4:**
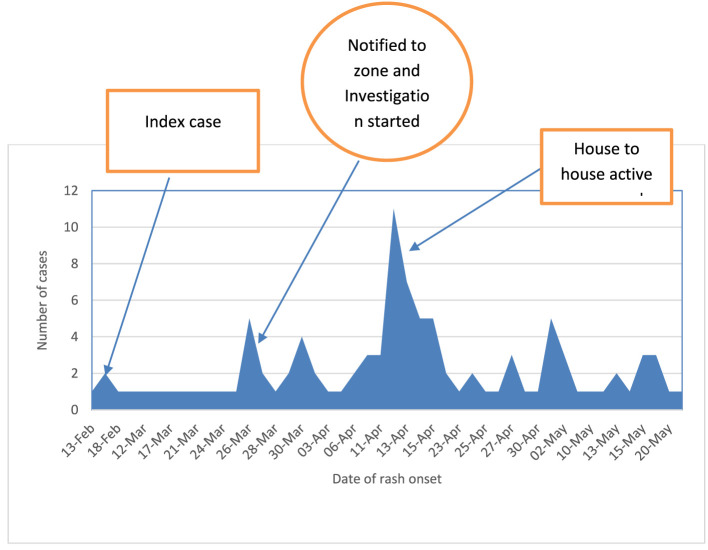
Epi Curve of the outbreak based on the date of onset of rash in Berhet district, Amhara region, Ethiopia, 2022.

On 27 February 2022, the initial case was observed at the Methbila health center, originating from Methbila kebele, 10 days after the onset of the rash. The index case, a 13-year-old female, had no record of vaccination. The patient had a travel history to a confirmed measles outbreak in an adjacent district before the onset of the rash. The patient did not experience any complications. After the index case, different cases were reported, and five samples were sent to the national laboratory for confirmation on 3 March 2022. The number of cases increased after active surveillance started. The case count experienced a swift rise from the initial week of April to the concluding week of April, as illustrated in Figure 4.

**Figure 5:**
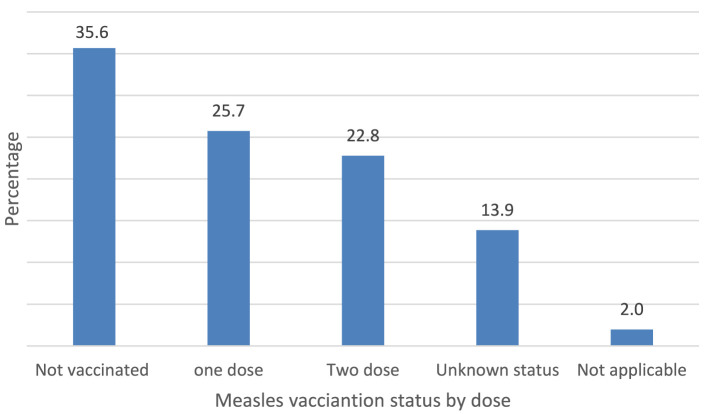
Vaccination status of measles cases in Berhet district, Amhara, Ethiopia, 2022.

Vaccination status of the cases: Out of the overall cases, 36 (35.6%) had not received the measles vaccine, while 14 (13.9%) either were unaware of their vaccination status or had missing information about it, as depicted in Figure 5.

### Vaccination coverage and cold chain management in the Berhet district

**Figure 6:**
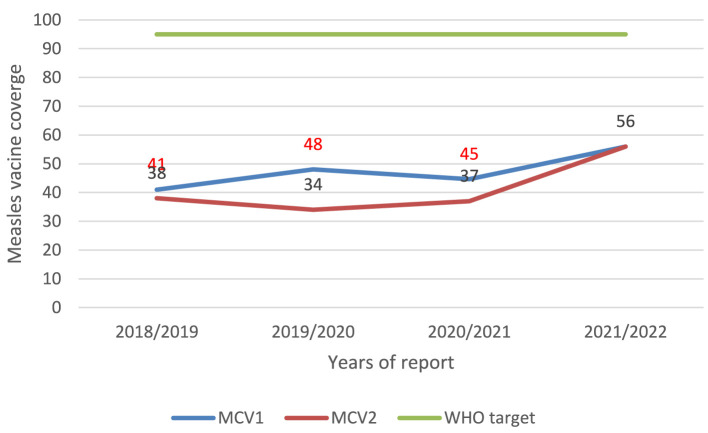
Coverage of MCV1 and MCV2 in Berhet district, North Shewa, Amhara region, 2022.

Throughout the outbreak investigation, we assessed the cold chain management of both the health office and health facilities in the Berhet district. The district health offices lack refrigeration, with all vaccines stored in the health center. However, each health center is equipped with a functioning fridge. Among the nine health posts, one does not have a functional fridge. Notably, the district lacks health workers trained in fridge maintenance, with only one EPI focal individual trained in cold chain management. Additionally, the administrative coverage of the district was below 60%, as indicated in Figure 6.

### Laboratory results

In March 2022, five blood samples were collected by laboratory technicians from individuals suspected of having measles in the Berhet district to validate the outbreak. Subsequently, these samples were sent to the laboratory at the Ethiopian Public Health Institute for confirmation of cases. Measles IgM antibodies were detected in one out of five samples; the other cases were epidemiologically connected to a known epidemic district nearby. Following the epidemiological and clinical declaration of a measles outbreak, five additional samples were sent to EPHI from various Methbila kebele goes for confirmation; four of the samples were negative, and one was positive for rubella IgM. Five more samples were submitted to confirm whether there had been an outbreak of rubella, but all came back negative. In total, 10 nasopharyngeal swabs were taken from Menjar district, the outbreak source district, to identify the virus strain, and the results revealed the presence of the measles virus strain in four samples.

### Analytic epidemiology

During the outbreak investigation, 60 measles cases and 120 recruited controls were asked a range of questions about measles transmission, prevention strategies, affected age groups, immunization status, contact history, travel history, home conditions, health-seeking behavior, and education level. Male participants made up 70% of the cases and 55% of the control group in the study. In terms of age distribution, 55% of controls and 66.1% of cases belonged to the age range of 5–15 years. Notably, 31.4% of cases and 26.9% of controls with vaccination history had a vaccination card. In total, 98% of the patients exhibited measles signs and symptoms. Six variables were found to be significant risk factors for contracting measles infection during the bivariate analysis. These variables included being male, not having received a measles vaccination, having traveled within 7–18 days before the onset of symptoms, having previously come into contact with someone who had the disease, not knowing that measles is a preventable illness, and not knowing the mode of transmission. Variables with p ≤ 0.20 were included in a multivariable logistic regression analysis to identify factors significantly associated with measles, using a significance threshold of < 0.05. Following adjustment for confounding effects in the multivariable model, five variables were identified as significantly associated with measles infection. Notably, a lack of vaccination against measles emerged as an independent risk factor for contracting the disease. A child's chance of contracting measles was 12 times higher among those who had not received the vaccination than in those who had [AOR = 12.06, 95% CI (3.12–46.52)]. People who had previously been to a location where measles was active were almost six times more likely to contract the illness than people who had never visited [AOR = 5.73, 95% CI (1.78–18.38)]. An independent risk factor for contracting measles was having contact with a patient who had the illness. When compared to those who had no contact history, the odds of contracting measles were ten times greater for those who had contact with someone who had the signs and symptoms of the illness [AOR = 10.3, 95% CI (3.48–30.5)]. Additionally, it was discovered that being aware of the measles' route of transmission protected against contracting the infection. People who knew the measles' mode of transmission, as learned from their mothers or caregivers, were 83.6% less likely to contract the illness than people who did not [AOR = 0.164, 95% CI (0.049–0.55)]. Similarly, 76.7% fewer people had measles than those who believed it was not a preventable disease. These people knew that measles was a preventable disease [AOR = 0.233, 95% CI (0.67–0.811)] (Table 1).

**Table 1:** Measles outbreak analysis, Berhet district, Amhara region, Ethiopia, May 2022.

**Variables**	**Measles status**		**Bivariable analysis**		**Multivariable analysis**	
	**Case N. (%)**	**Control N. (%)**	**COR (95% CI)**	***P*** **-value**	**AOR (95% CI)**	***P*** **-value**
**Sex**						
Female	18 (30%)	54 (45%)	0.524 (0.271–1.012)	0.054^*^	0.349 (0.115–1.064)	0.064
Male	42 (70%)	66 (55%)	1		1	
**Age**						
< 1 year	1 (1.7%)	1 (0.8%)	1			
1–4 years	7 (11.7%)	12 (10%)	0.583 (0.031–10.8)	0.718		
5–14 years	40 (66.7%)	66 (55%)	0.606 (0.037–9.96)	0.726		
≥15 years	12 (20%)	41 (34.2%)	0.293 (0.17–5.03)	0.397		
**Distance from a health facility**						
< 5 km	38 (63.3%)	74 (61.7%)	1			
>5 km	22 (36.7%)	46 (38.3%)	0.931 (0.49–1.76)	0.828		
**Vaccination status**						
Yes	21 (35%)	63 (52.5%)	1		1	
No	23 (38.3%)	11 (52.5%)	6.273 (2.6–15)	0.000^*^	12.06 (3.12–46.52)	0.000^*^
Unknown status	16 (26.7%)	46 (38.3%)	0.912 (1.04–0.94)	0.912	0.851 (0.27–2.6)	0.782
**Travel time (7–18 days)**						
Yes	52 (86.7%)	52 (43.3%)	8.5 (3.7–19.4)	0.000^*^	5.73 (1.78–18.38)	0.003^*^
No	8 (13.3%)	68 (56.7%)	1			
**Contact history**						
Yes	53 (88.3%)	31 (25.8%)	21.7 (8.9–52.8)	0.000^*^	10.3 (3.48–30.5)	0.000^*^
No	7 (11.7%)	89 (74.2%)	1			
**Do you think measles is preventable?**						
Yes	37 (61.7%)	108 (90%)	0.179 (0.81–0.394)	0.000^*^	0.233 (0.67–0.811)	0.022^*^
No	23 (38.3%)	12 (10%)	1			
**Know the transmission mood of measles**						
Yes	31 (51.7%)	110 (91.7%)	0.097 (0.43–0.221)	0.000^*^	0.164 (0.049–0.55)	0.003^*^
No	29 (48.3%)	10 (8.3%)	1			

Unknown status: Either the individuals affected by the outbreak have not been vaccinated against measles, or their vaccination status is unknown due to incomplete records or other factors. In bivariable analysis ^*^(*p*-value < 0.20), while in multivariable analysis ^*^(*p*-value < 0.05).

### Outbreak response

Following the announcement of a measles outbreak in the Berhet district, the district health office of Berhet convened an emergency meeting, provided onsite orientation, assigned a team to assist the primary health facility rapid response team, assessed the situation, improved the active case search, provided the standard format for a measles line-list, and reviewed the available resources to determine whether more were needed. Alerts were sent out, and the reporting kebele was asked to provide more information to the fast-reaction team. The district health office, in collaboration with the health center's RRT and HEW, actively searched for cases of measles in local communities, schools, religious institutions, and private clinics. The assessment team from the North Shewa Zone Health Department was then dispatched to the Berhet District Health Office to investigate the incident. The district health officer and the outbreak investigation team met and discussed how to execute measles outbreak prevention and control. Approximately 20 cases of measles were found in the neighborhoods during an inquiry and active surveillance.

### Case management

All suspected cases were treated using vitamin A capsules. One dose was given at the time of diagnosis, and for adult patients, the second dose was given to take the next day, with the last dose administered by day 14. Antibiotics were administered to treat carriers through house-to-house visits based on their clinical manifestations. Community mobilization activities were carried out in collaboration with local community representatives and kebele leaders to encourage routine vaccine uptake. An active case search was conducted in the community by staff members of the health center, health post, zonal, and district health departments to prevent the spread of the disease to other kebeles. Health centers received vitamin A to treat patients without charging for their care, and all medical staff received technical support for managing cases, documenting information, and reporting findings. To halt the spread of the measles outbreak and reduce morbidity, cases were treated.

A district-wide mass vaccination campaign was launched for high-risk age groups, including 9 months to 2 years and up to 15 years, in Methbila kebele, the area with the highest measles incidence.

## Discussion

This study aimed to delineate the extent of the measles outbreak and identify the factors contributing to disease contraction in the Berhet district. After detecting three serum samples with positive measles-specific IgM antibodies in the neighboring district, along with one confirmed measles case and other cases linked epidemiologically to the laboratory-confirmed ones, the outbreak was officially declared. The results of the descriptive analysis demonstrate that the measles outbreak had an overall attack rate of 20 cases per 10,000 people, and the age group most affected was from 5 to 15 years. We found a lower attack rate than the attack rate of the measles outbreak investigation conducted in the Ginner district, 63/10,000 (13), and higher than a study in the Kabridehar district of Somali Regional State, Ethiopia, which reported an AR of 4/10,000 (14), and a study in Guji zone, Oromia region, 8.1/10,000 (15). The concentration of vulnerable individuals likely accelerated the spread of the disease, leading to a higher attack rate. Additionally, the district's vaccination rates for MCV1 and MCV2 have been below 60% for the past 3 years due to low vaccination rates. The widespread, high attack rates across a larger age range may indicate that routine immunization rates have been consistently low for a number of years, which may have contributed to the present outbreak. This conclusion implies the need for routine immunization and supplementary immunization activities (SIAs), as well as for monitoring the buildup of susceptible persons, to safeguard both target and non-target age groups (16). No fatalities have been documented in this outbreak. The current case fatality rate (CFR) estimates utilized by the World Health Organization (WHO) in low-income countries span from 0.05% to 6% (17). In complex emergencies or isolated areas where there is either low natural immunity or low vaccination coverage, the CFR is often between 10% and 30% (18, 19). This zero CFR observed in our study was totally different from different findings of measles outbreak investigations conducted in Ethiopia (3, 13, 15, 20). It is possible that early reactions and case management were implemented in the impacted kebeles, which is why this study had no fatalities. It could also be because deaths that occur within the community go unreported. A child's chance of contracting measles was 12 times higher for those who had not received the vaccination compared to those who had. This is consistent with the results of a case-control study carried out in the Somali Regional State, Ethiopia, in the districts of Kabridehar and Kabridehar town, where it was found that having received the measles vaccine protects against contracting the disease (3, 14, 21).

Likewise, our results align with a case-control study conducted in Uganda, suggesting that the lack of measles vaccination was a primary factor contributing to most measles infections in children (22). This is because measles vaccination is crucial for preventing measles infection (23, 24). Achieving 95% population immunity is essential to prevent measles outbreaks, disrupt transmission, and promote the elimination of measles (7). This discovery implies that the measles outbreak may be attributed to the accumulation of individuals susceptible to measles infection. Individuals with a history of traveling to regions with active measles were six times more likely to contract measles compared to those who did not travel. This observation is corroborated by comparable studies conducted in the Bale zone, Oromia, Ethiopia, and in the Ginner district (13, 20).

This arises from either direct contact with an infected individual or an elevated risk of contracting the disease in an area currently experiencing measles activity. Another distinct risk factor for measles acquisition was exposure to an individual already diagnosed with the illness. Those who had direct contact with a measles case patient faced an almost tenfold higher risk of contracting the disease compared to those with no prior contact. This observation is substantiated by the findings of a case-control study conducted during an outbreak investigation in Southwest Ethiopia and by other studies in various rural districts of Ethiopia (13, 15, 20, 21, 25) and other similar study done in Uganda (9), showing the likelihood of contracting the disease was more than three times higher when in touch with a case. This is due to the transmission of measles through respiratory droplets or direct/indirect contact with the nasal and throat secretions of infected individuals. Moreover, the secondary attack rate for measles surpasses 90% in the presence of susceptible individuals (6).

It has been discovered that mothers' and caregivers' knowledge about measles transmission protects against contracting the illness. The findings align with a study conducted in the Artuma Fursi Oromia zone of the Amhara region of Ethiopia. In that study, it was observed that women who recognized the importance of measles vaccination were three times more likely to immunize their children compared to mothers who were not aware of the vaccine (25). A study in ten high-burden countries shows similar findings (23). Furthermore, the research carried out in Southwest Ethiopia indicated that mothers with sufficient knowledge of preventive measures against measles were at a reduced risk of contracting the infection (26).

## Limitations of the study

Most individuals did not have vaccination records. We asked the participants or caregivers whether their children had received the measles vaccination at 9 months of age or older on the upper left arm. The lack of a vaccination card made it difficult to ascertain the precise date and status of immunization, which is likely why recall bias exists.

## Conclusion and recommendations

The outbreak exhibited a higher incidence rate, affecting a wider age range, predominantly children aged 5–14 years. More than 35% of cases lacked measles vaccination, revealing a low administrative vaccination rate. Several factors contributed to the outbreak, including non-vaccination, travel to areas with active measles, contact with cases, and insufficient knowledge among mothers and caregivers about measles transmission and prevention. To address this, efforts should focus on reaching remote areas, enhancing surveillance, supporting community immunization drives, and establishing isolation facilities. The vaccination program should extend measles immunization up to the age of 15 years, coordinated by the APHI and regional health authorities.

## Data availability statement

The raw data supporting the conclusions of this article will be made available by the authors, without undue reservation.

## Author contributions

YS: Visualization, Data curation, Writing – review & editing, Methodology, Formal analysis, Conceptualization. AA: Writing – original draft, Project administration, Visualization, Methodology, Formal analysis, Data curation, Conceptualization. TT: Writing – original draft, Resources, Methodology, Formal analysis, Data curation, Conceptualization. SF: Writing – review & editing, Writing – original draft, Methodology, Formal Analysis, Conceptualization.
